# Female reproductive factors are associated with objectively measured physical activity in middle-aged women

**DOI:** 10.1371/journal.pone.0172054

**Published:** 2017-02-22

**Authors:** Eija K. Laakkonen, Janne Kulmala, Pauliina Aukee, Harto Hakonen, Urho M. Kujala, Dawn A. Lowe, Vuokko Kovanen, Tuija Tammelin, Sarianna Sipilä

**Affiliations:** 1 Gerontology Research Center, Faculty of Sport and Health Sciences, University of Jyväskylä, Jyväskylä, Finland; 2 Department of Rehabilitation Medicine, Divisions of Rehabilitation Science and Physical Therapy, Medical School, University of Minnesota, Minneapolis, Minnesota, United States of America; 3 LIKES Research Centre for Physical Activity and Health, Jyväskylä, Finland; 4 Department of Obstetrics and Gynecology, Pelvic Floor Research and Therapy Unit, Central Finland Central Hospital, Jyväskylä, Finland; 5 Faculty of Sport and Health Sciences, University of Jyväskylä, Jyväskylä, Finland; Victoria University, AUSTRALIA

## Abstract

Physical activity improves health and may delay the onset of several chronic diseases. For women in particular, the rate of these diseases accelerates at middle age; therefore it is important to identify the determinants of health-enhancing physical activity during midlife in this population. In this study, we focused on determinants that are unique to the female sex, such as childbearing and menopause. The main objective was to characterize the level of physical activity and differences between active and inactive middle-aged Finnish women. In addition, we examined the association of physical activity with female reproductive factors at midlife. The study population consisted of 647 women aged 48 to 55 years who participated in our Estrogenic Regulation of Muscle Apoptosis (ERMA) study during the period from 2015 to 2016. Physical activity was measured objectively using hip-worn accelerometers for seven consecutive days. The outcome measures included the amounts of light intensity physical activity and moderate to vigorous intensity physical activity accumulated in bouts of at least 10 minutes (MVPA_10_). MVPA_10_ was used to determine whether women were placed in the active (≥150 min/week) or inactive (<150 min/week) group. Multiple linear regression models were performed with physical activity measures as dependent variables and cumulative reproductive history index, menopausal symptoms, and pelvic floor dysfunction as independent variables. We found that a large portion (61%) of Finnish middle-aged women did not meet the physical activity recommendations of 150 minutes of MVPA_10_ per week. In the studied cohort, 78% of women experienced menopausal symptoms, and 54% exhibited pelvic floor dysfunction. Perceived menopausal symptoms were associated with greater light physical activity. Perceived pelvic floor dysfunction was associated with lower MVPA_10_. According to the fully adjusted multiple linear regression models, reproductive factors explained 6.0% of the variation of MVPA_10_ and 7.5% of the variation of light physical activity. The results increase our knowledge of the factors related to physical activity participation among middle-aged women and indicate that menopausal symptoms and pelvic floor dysfunction should be identified and considered when promoting physical activity for women during midlife. The results emphasize that awareness of female reproductive factors, especially menopausal symptoms and pelvic floor dysfunction, is important for physical activity counseling to effectively help women in performing and sustaining health-enhancing amounts of physical activity. Specifically, the condition of the pelvic floor should be taken into account when identifying the proper activity type and intensity level so that health benefits of physical activity can still be attained without worsening symptoms.

## Introduction

It is well-recognized that sedentary behavior predisposes individuals to adverse health conditions, while physical activity counteracts many of these adversities [1]. The World Health Organization (WHO) recommends at least 150 minutes per week of moderate to vigorous intensity physical activities (MVPA) or 75 minutes per week of vigorous intensity physical activities to achieve health benefits [2]. Moreover, MPVA is recommended to be accumulated as bouts lasting at least 10 minutes (MVPA_10_) [2]. Despite the guidelines, only a fraction of the world population complies with these recommendations [3]. Furthermore, the latest world-wide estimates, based primarily on observational associations, show that approximately 9% of premature deaths are caused by physical inactivity [4]. In addition to MVPA_10_, shorter physical activity bouts may produce health benefits [5]. Recently, total physical activity counts including light and intermittent MVPA were found to have a stronger association with insulin resistance than MVPA_10_ [6], and greater light physical activity was shown to be associated with lower mortality risk among less active persons [7]. However, the association between low intensity activities and being overweight or obese was found to be weak [8]. Thus, more research on the benefits of physical activity performed at different intensities and durations are warranted. Furthermore, sex-specific physical activity recommendations are lacking and sex-specific knowledge on the intensity and amount of physical activity participation is sparse. Thus, identifying potential reasons for inadequate physical activity, especially among middle-aged women, is important to promote health in this sub-population.

Female reproductive factors, such as early menarche, high number of gestations, high parity, miscarriages, and early menopause, are associated with low cardiovascular health [9]. The adverse health association of these individual reproductive factors may be mediated by the cumulative association with low physical activity. For instance, high parity has been associated with another common barrier of physical activity, lack of time [10,11], and is also a risk for pelvic floor dysfunction. Pelvic floor dysfunction in turn is associated with low physical activity [12,13]. Thus, it may be that a variety of female reproduction-associated life events ranging from menarche to menopause form cumulative barriers predisposing middle-aged women to be physically inactive.

Physical activity has been investigated as a potential remedy for menopausal symptoms with conflicting results (see the recent review by Grindler and Santoro [14]), but there is a lack of research on menopausal symptoms as potential reasons for being physically active or inactive. On the other hand, a physically inactive lifestyle has been associated with more severe menopausal symptoms [15] and with more deleterious psychological, somatic, and vasomotor symptoms [16]. Therefore, it is reasonable to postulate that typical menopausal symptoms may modulate physical activity behavior in women. However, it may also be possible that physical activity behavior may modulate the severity of menopausal symptoms.

Pelvic floor dysfunction is a common condition that includes any combination of the following symptoms: urinary or fecal incontinence, constipation or defecation difficulties, and pelvic organ prolapse [17]. Pelvic floor dysfunction, especially urinary incontinence, is one of the few medical conditions for which high-impact physical activity may be a predisposing factor [12,18,19]. Additional risk factors for pelvic floor dysfunction include age, obesity, diabetes, gestations, parity, and estrogen deficiency [13]. As summarized in a recent review article, a limited number of studies have investigated the association between pelvic floor dysfunction and physical activity among women, and most have focused on urinary incontinence [17]. The pioneering survey of more than 41,000 Australian women aged 18 to 75 years by Brown and Miller showed that 7% of younger, more than 33% of middle-aged, and 25% of older women reported the avoidance of sporting activities because of urinary incontinence [20]. However, there are no studies in which the amount of physical activity was measured objectively and related to specific symptoms of pelvic floor dysfunction in the age group most affected by the condition, namely middle-aged women.

The aim of this study was to characterize the level of physical activity and potential group differences between physically active and inactive 48 to 55-year-old Finnish women. We examined the association between female reproductive factors and objectively-measured physical activity. We hypothesized that cumulative reproductive history index, perceived menopausal symptoms, or pelvic floor dysfunction are associated with low levels of physical activity in middle-aged women.

## Materials and methods

### Study design and participants

**Fig 1:**
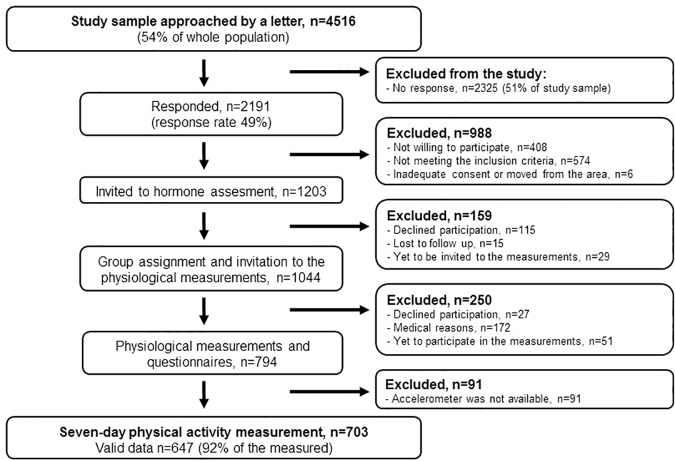
Flow diagram of the recruitment of study participants.

This study uses data that was collected in the Estrogenic Regulation of Muscle Apoptosis (ERMA) -study, which investigates health, functional capacity, muscle strength, and underlying biological mechanisms of skeletal muscle among 48 to 55-year-old women living in the city of Jyväskylä or neighboring municipalities in Finland (Fig 1). This paper reports results from participants assessed before the 7^th^ of April, 2016, representing a random sample of the target population registered in the Finnish National Registry and randomly selected by Registry authorities using equally-spaced sampling (54% of the entire age cohort). Participants were contacted by written invitation to participate in the study (n = 4516). Exclusion criteria included a self-reported body mass index (BMI) >35 kg/m^2^, being currently pregnant or lactating, polycystic ovary syndrome or other conditions affecting ovarian function, bilateral ovariectomy, estrogen containing hormonal preparations or other medications affecting ovarian function, and chronic diseases or medications seriously affecting muscle function (excluded n = 988). No response was received from 2325 women, leading to a response rate of 49%. The eligible participants who responded (n = 1203) were invited to the laboratory for blood sampling and for a structured interview about their current medical condition. Those considered eligible and willing to participate in the study were invited to a second laboratory visit for physiological measurements (n = 794). The dataset reported in the current study consists of the 647 participants who provided acceptable accelerometer data, representing 92% of the participants who wore accelerometers.

All study participants provided written informed consent. The study protocol followed good clinical and scientific practice and the Declaration of Helsinki and was approved by the ethics committee of the Central Finland Health Care District (K-S shp Dnro 8U/2014).

### Objective measurement of physical activity

Physical activity was assessed with GT3X+ and wGT3X+ Actigraph accelerometers (Pensacola, Florida, USA). Each participant was personally instructed regarding how to use Actigraph accelerometric devices and was provided with an accompanying diary. Participants were instructed to wear monitors on the right hip during waking hours for seven consecutive days except while bathing or doing other water-based activities. They were instructed to record, in their diary, their waking and working hours as well as periods when they needed to remove the accelerometer for more than 30 min. Raw acceleration data was collected at 60 Hz and was filtered and converted into 60 s epoch counts. Tri-axial vector magnitude was computed as the composite vector magnitude of three individual orthogonal planes to accommodate cut-points for tri-axial vector magnitude in order to distinguish the intensity of physical activity as sedentary time versus light, moderate, or vigorous physical activity [21].

Sasaki et al. (2011) used tri-axial vector magnitude cut-points of 2690 and 6166 counts per minute (cpm) to separate moderate and vigorous intensity physical activity, respectively [21]. To our knowledge, the use of cut-points to distinguish sedentary from light physical activity has not been reported for adults. However, 100 cpm is widely used as a cut-point between sedentary and light physical activity in analyses of vertical axis only (as opposed to tri-axial vectors). Therefore, sedentary time was computed from the vertical axis with the widely used 100 cpm and then compared to sedentary time analyzed from the tri-axial vector data with several cut-points (**S1 Table**). 450 cpm correlated highly with 100 cpm in vertical axis analyses (r = 0.96, mean difference -2 min/day) and was chosen to supplement the cut-points used by Sasaki et al. (i.e., sedentary ≤ 450 cpm, light >450 to ≤2690 cpm; moderate >2690 to ≤6166 cpm; vigorous >6166 cpm) [21]. Comparisons of tri-axial and vertical axis analysis for these different physical activity intensities are presented in **S2 Table**.

A customized Excel-based program was used for further data analysis. For a valid monitoring period, 600 min/day or more and at least 4 days of measured time were required [22]. Non-wear time was defined as periods of 60 min of consecutive zero counts. In vertical axis analyses, 20,000 cpm is considered to be non-human produced [22] and as such, spurious acceleration of 25,000 cpm was set as the upper limit for our tri-axial data.

The number of steps, mean time spent at different physical activity intensities per day, and MVPA were calculated for each participant. Sedentary time was calculated as a percentage of total accelerometer wearing time. MVPA time was also examined in bouts. Bouts of MVPA (MVPA_10_) were defined as those with ≥10 consecutive minutes of MVPA [23] with an allowance of a 1 min interruption per 5 min.

Study participants were divided into physically active and inactive groups according to the WHO recommendation [2] and based on calculated MVPA_10_. Women were considered as physically active if they had at least 150 minutes per week of MVPA_10_ and inactive if they had less than 150 minutes MVPA_10_ per week.

### Reproductive factors

In the current study, self-reported data on age at menarche, parity and gestations were collected. Miscarriages were calculated as differences between gestations and parity.

Based on bleeding diaries and serum concentrations of follicle stimulating hormone (FSH) and 17β-estradiol (E_2_) (Immulite; Siemens), participants were categorized as premenopausal (E_2_ = 0.5±0.7 nmol/l, FSH = 8.6±3.7 IU/l), perimenopausal (E_2_ = 0.3±0.2 nmol/l, FSH = 34.9±22.0 IU/l) or postmenopausal (E_2_ = 0.1±0.1 nmol/l, FSH = 82.4±30.0 IU/l) following the STRAW +10 guidelines [24]. Serum samples to measure hormone concentrations were obtained during menstrual days 1–5 as follows; whole blood was collected under standard fasting conditions from the antecubital vein while the participant was in a supine position. Serum was separated by centrifugation for 10 minutes at 2200 x g. Women who had undergone a hysterectomy or who used hormonal intrauterine devices (n = 262), were classified solely based on their hormonal status (premenopausal n = 93, perimenopausal n = 70, and postmenopausal n = 99).

Factors of reproductive history, including early menarche, high parity, miscarriages and being postmenopausal, as a cumulative index have been shown to be related to adverse health outcomes [9]. Therefore, a cumulative reproductive history index ranging from 0 to 4 was calculated with each of the following counting as a score of one: age at menarche less than 12, parity ≥3, one or more miscarriages, and being postmenopausal. This index was used in the regression analysis as a predictive variable.

### Self-reported menopausal symptoms

Menopausal symptoms were recorded using a structured questionnaire. Participants were asked to indicate if they had any of the following ten symptoms: sweating, hot flashes, sleeping problems, headache, joint pain, tiredness, mood swings, vaginal symptoms, urinary track problems, and sexual problems. The questionnaire also included the option to describe a maximum of three additional symptoms. Based on responses, four menopausal symptom clusters were formed: Vasomotor symptoms (sweating, hot flushes, cold flushes, heart palpations and coldness); Somatic or pain symptoms (headache, joint pain, stomach pain, migraine, hip pain, muscle pain, breast pain, dizziness, swelling, weakness); Psychological symptoms (sleeping problems, tiredness, mood swings, memory problems, irritability, inability to concentrate, weepiness); and Urogenital symptoms (vaginal symptoms, urinary track problems, sexual problems, vaginal infection, urinary tract infection, and vaginal dryness) [15,16]. The number of reported menopausal symptom classes was tested as a predictive variable in the regression models.

### Self-reported symptoms of pelvic floor dysfunction

The occurrence of stress urinary incontinence, urge or urgency urinary incontinence, fecal incontinence, constipation or defecation problems, and feeling of pelvic organ prolapse within the previous month were assessed by questionnaire. The specific questions were the following: Have you had within the last month urinary incontinence during physical effort or coughing? Have you had within last the month urge or urgency-related urinary incontinence? Have you had within the last month fecal incontinence? Have you had within the last month constipation or defecation difficulties? Have you had within the last month a feeling that something is falling out of the vagina? A cumulative pelvic floor dysfunction index (zero, one, two or more dysfunction types) was formed in order to capture potential additive effects of having more than one symptom at the same time. This index was used as a predictive variable in the regression models.

### Socioeconomic and lifestyle variables

Level of education was classified as lower (primary and secondary school degrees) or higher (bachelor and higher degrees) level. Marital status was used to determine if the participant lived with a partner (married, common-law, or registered partnership). Employment status was conceptualized as a dichotomous variable in which participants were either employed (paid or self-employed) or not regularly employed (student, unemployed, working occasionally, retired, taking care of home). As an indicator of general health, self-reported chronic conditions as diagnosed by a physician were recorded. The number of conditions was used to describe the general health status of the participants. BMI was calculated as weight/squared height (kg/m^2^), and participants were categorized into normal weight (BMI≤25 kg/m^2^), overweight (BMI 25–30 kg/m^2^), or obese (BMI≥30 kg/m^2^). Participants were categorized according to their smoking history and habits as never, quitter, or current smoker. Never smokers included women who reported smoking less than 10 packs of cigarettes during their life. Participants drinking habits were categorized as never or rarely if they reported consuming less than 1 portion/week, weekly if they reported having 1–5 portions/week, and often if they reported having more than 5 portions/week.

### Statistics

Normality of variables was assessed with the Shapiro Wilks Test, and skewed data were analyzed using the Mann-Whitney U rank test. A χ^2^ -test was used for categorical variables and the Mann-Whitney U test for continuous variables to test statistical significance between the physically active and inactive groups. Multivariate linear regression models were constructed to estimate the association of cumulative reproductive history index, number of menopausal symptoms, and number of pelvic floor dysfunction with MVPA_10_ or light physical activity. Since it is known that physical activity can also be affected by socioeconomic and lifestyle factors, we assessed potential confounding factors by determining how much the regression coefficient of the independent variable changed after the addition of the potential confounder to the model. Factors that caused a 9.4% or greater change in the regression coefficient of the predictor were considered as confounders and were added to the final models [25]. Variables tested as potential confounders were education, marital status, employment status, chronic diseases, BMI, smoking, and alcohol consumption (**S3 Table**). Model 1 is an unadjusted model. Confounders were included in the models step-by-step. Data analyses were carried out using IBM SPSS Statistics 22 (SPSS Inc., Chicago, IL). The level of significance was set at p≤0.05.

## Results

### Descriptive characteristics and physical activity in middle-aged women

In total, 39% of the participants were physically active based on MVPA_10_ ≥150 minutes per week (**Table 1**). There were no significant differences in age, education, marital status, employment status, or number of chronic diseases between physically active and inactive groups. Fewer physically active women were overweight or obese, and a greater percentage were normal weight compared with women in the inactive group. There was no group difference in alcohol consumption. The percentage of current smokers was lower and the percentage of quitters was greater in the physically active than the inactive group. The mean number of acceptable accelerometer measurement days was 6.8, and the mean wearing time was more than 15 hours per day for both groups. Mean daily steps and time spent doing moderate and vigorous physical activity were higher for women in the physically active group compared to those in the inactive group, while the groups did not differ in their amount of light physical activity. There was a 2.3% mean difference in the relative time being sedentary between the physically active and inactive women.

**Table 1:** General characteristics and physical activity of women in the physically active and inactive groups.

	Physically active^§^ n = 251 (39%)	Physically inactive^§^ n = 396 (61%)	p-value
**Age in** years: mean (SD)	51.4 (2.1)	51.5 (2.0)	0.288
**Education:** % [n]			0.501
Lower level	59.0 [148]	61.6 [244]	
Higher level	41.0 [103]	38.4 [152]	
**Marital status:** % [n]^#^			0.173
Lives with a partner	72.0 [180]	76.8 [304]	
Lives without a partner	28.0 [70]	23.2[92]	
**Employment status:** % [n]			0.501
Employed	85.3 [214]	87.1 [345]	
Not regularly employed	14.7 [37]	12.9 [51]	
**# of chronic diseases**: % [n]			0.090
0	47.0 [118]	42.9 [170]	
1	38.6 [97]	35.9 [142]	
2 or more	14.3 [36]	21.2 [84]	
**Weight categories:** % [n]			**<0.001**
Normal weight (BMI<24.9 kg/m^2^)	64.5 [162]	44.2 [175]	
Overweight (25<BMI<30 kg/m^2^)	29.1 [73]	38.1 [151]	
Obese (BMI>30 kg/m^2^)	6.4 [16]	17.7 [70]	
**Smoking:** % [n]			**0.026**
Never	71.7 [180]	72.2 [286]	
Quitter	23.9 [60]	18.4 [73]	
Smoker	4.4 [11]	9.3 [37]	
**Alcohol consumption:** % [n]^#^			0.863
Never or rarely (<1 portion/week)	45.0 [113]	43.5 [172]	
Weekly (1–5 portions/week)	43.4 [109]	45.6 [180]	
Often (>5 portions/week)	11.6 [29]	10.9 [43]	
**Objectively measured physical activity:**mean (SD)			
# of acceptable measurement days	6.9 (0.4)	6.8 (0.5)	0.062
Daily accelerometer wearing time (min)	919.6 (71.3)	912.4 (67.4)	0.519
% of working time from total measurement time	24.5 (16.1)	25.7 (16.1)	0.538
Mean daily activity counts (counts/min)	773.7 (188.0)	605.1 (167.7)	**<0.001**
Mean daily steps	10623 (2643)	7479 (2241)	**<0.001**
Sedentary time (%)	60.9 (8.8)	63.2 (9.4)	**0.002**
Light PA (min/day)	290.6 (76.0)	297.4 (78.0)	0.287
Moderate PA (min/day)	58.2 (22.1)	35.8 (17.6)	**<0.001**
Vigorous PA (min/day)	10.7 (11.3)	1.8 (2.9)	**<0.001**
MVPA (min/day)	68.9 (23.8)	37.7 (18.2)	**<0.001**
MVPA_10_ (min/day)	37.5 (14.9)	9.1 (6.4)	**<0.001**

χ^2^ -test was used for categorical variables and Mann-Whitney U test for continuous variables.

^§^ Physically active group has MVPA_10_ ≥150 min/week and inactive group has <150 min/week.

^#^ missing or unclear data: marital status n = 1, and alcohol consumption n = 1. BMI = body mass index, PA = physical activity, MVPA = moderate to vigorous intensity physical activities, and MVPA_10_ = cumulative time on at least 10-minute activity bouts performed at moderate to vigorous intensity. In the case of mean values, numbers in the rounded parentheses are standard deviations, while for percentages, numbers in the squared parentheses are numbers of participants.

### Reproductive history, menopausal symptoms and pelvic floor dysfunction in physically active and inactive women

Most factors of reproductive history were not different between the physically active and physically inactive groups (**Table 2**). The mean age of first menstruation was 13 years in both groups, and the means for number of gestations were 2.6 and 2.5 in the active and inactive groups, respectively. Physically active women were more likely to be nulliparous, but also had a greater occurrence of having three or more children, compared to the inactive women, who were more likely to have one or two children. There were no statistically significant differences between physically active and inactive women in the incidence of miscarriages, the level of circulating E_2_ and FSH, the percentage of pre-, peri- or postmenopausal women, or the cumulative reproductive history index.

**Table 2:** Characteristics associated with female reproductive biology.

	Physically active^§^ n = 251	Physically inactive^§^ n = 396	p-value
**Age in years at first menstruation:** mean (SD)^#^	13.1 (1.3)	13.0 (1.3)	0.470
**Number of gestations:** mean (SD)^#^	2.6 (1.8)	2.5 (1.5)	0.202
**Number of children:** mean (SD)^#^	2.1 (1.5)	2.0 (1–2)	0.766
**Gestations**: % [n]^#^			**<0.001**
None	12.4 [31]	7.9 [31]	
One or two	33.3 [83]	48.9 [192]	
Three or more	54.2 [135]	43.3 [170]	
**Parity: % [n]**			**0.023**
Nulliparous	16.0 [40]	10.6 [42]	
One or two	49.2 [123]	59.5 [235]	
Three or more	34.8 [87]	29.9 [118]	
**If been pregnant, number of terminations/miscarriages:** % [n]^#^			0.055
None	61.8 [154]	70.0 [275]	
One or two	35.3 [88]	26.5 [104]	
Three or more	2.8 [7]	3.6 [14]	
**Circulating hormones:** mean (SD)			
17β-estradiol (nmol/l)	0.3 (0.3)	0.3 (0.4)	0.408
Follicle stimulating hormone (IU/l)	50.9 (44.2)	44.4 (35.9)	0.111
**Menopausal status:** % [n]			0.503
Premenopausal	25.9 [65]	28.8 [114]	
Perimenopausal	30.7 [77]	32.3 [128]	
Postmenopausal	43.4 [109]	38.9 [154]	
**Cumulative reproductive history index:** % [n]^#^			0.063
None	22.9 [57]	26.8 [104]	
One or two	30.7 [77]	67.5 [262]	
Three to four	43.4 [109]	5.7 [22]	
**Menopausal symptoms:** % [n]^#^			
Any symptoms	79.3 [199]	77.5 [306]	0.586
Vasomotor symptoms	63.3 [159]	60.8 [240]	0.510
Somatic/Pain symptoms	23.5 [59]	25.6 [101]	0.554
Psychological symptoms	47.8 [120]	50.9 [201]	0.446
Urogenital symptoms	35.1 [88]	37.0 [146]	0.624
**Number of reported menopausal symptom classes:** % [n]^#^			0.420
None	20.7 [52]	22.5 [89]	
One or two	50.6 [127]	45.3 [179]	
Three to four	28.7 [72]	32.2 [127]	
**Pelvic floor dysfunction:** % [n]			
Any type of dysfunction: % [n]	43.8 [110]	60.1 [238]	**<0.001**
Stress urinary incontinence: % [n] ^#^	31.9 [80]	43.1 [170]	**0.004**
Urge or urgency urinary incontinence: % [n] ^#^	14.3 [36]	16.3 [64]	0.498
Fecal incontinence: % [n]^#^	2.0 [5]	4.1 [16]	0.151
Constipation or defecation problems: % [n]	13.6 [34]	18.0 [71]	0.139
Feeling of pelvic organ prolapse: % [n]	4.4 [11]	5.8 [23]	0.427
**Number of pelvic floor dysfunctions:** % [n]			**<0.001**
None	56.2 [141]	39.9 [158]	
One	24.7 [62]	40.4 [160]	
Two or more	19.1 [48]	19.7 [78]	

Although a high number of participants (78%) reported menopausal symptoms, there were no significant differences reported for the number of menopausal symptoms between physically active and inactive women. In the entire cohort, 54% reported to have some type of pelvic floor dysfunction. The percentage of women with dysfunction was smaller in the active than inactive group. The most common type of pelvic floor dysfunction was stress urinary incontinence, which was reported by 32% of physically active women and 43% of inactive women. According to the cumulative index for pelvic floor dysfunction, a larger portion of the active women was free of pelvic floor dysfunction compared to the inactive women, while a larger portion of inactive women had at least one type of pelvic floor dysfunction compared to the active women.

### Female reproductive factors and physical activity

**Table 3** shows multiple linear regression analysis of MVPA_10_ as the dependent variable and **Table 4** shows the same analysis for the total time spent on light physical activity. The number of pelvic floor dysfunctions was negatively associated with MVPA_10_; although, after adjusting for chronic diseases and smoking (models 4 and 5) the p-value shifted to non-significant. The number of menopausal symptoms and cumulative reproductive history index were not significantly associated with MVPA_10_. The fully adjusted model explained 6.0% of the variation of MVPA_10_.

**Table 3:** Multiple linear regression analysis of at least 10-minute activity bouts performed at moderate to vigorous intensity (MVPA_10_) as dependent variable.

	MVPA_10_			model	
	B (SE)	Beta	p-value	R^2^	p-value
**Model 1**				0.016	**0.019**
Cumulative reproductive history index	1.365 (0.953)	0.058	0.152		
Number of menopausal symptoms	-0.511 (0.623)	-0.033	0.412		
Number of pelvic floor dysfunctions	-2.362 (0.906)	-0.104	**0.009**		
**Model 2: adjusted with BMI**				0.054	**<0.001**
Cumulative reproductive history index	1.589 (0.936)	0.067	0.090		
Number of menopausal symptoms	-0.535 (0.611)	-0.035	0.382		
Number of pelvic floor dysfunctions	-1.930 (0.893)	-0.085	**0.031**		
**Model 3: as 4 and education**				0.055	**<0.001**
Cumulative reproductive history index	1.654 (0.938)	0.070	0.078		
Number of menopausal symptoms	-0.540 (0.611)	-0.035	0.377		
Number of pelvic floor dysfunctions	-1.867 (0.895)	-0.082	**0.037**		
**Model 4: as 3 and chronic diseases**				0.056	**<0.001**
Cumulative reproductive history index	1.639 (0.939)	0.069	0.081		
Number of menopausal symptoms	-0.494 (0.613)	-0.032	0.421		
Number of pelvic floor dysfunctions	-1.747 (0.907)	-0.077	**0.054**		
**Model 5: as 4 and smoking**				0.060	**<0.001**
Cumulative reproductive history index	1.728 (0.940)	0.073	0.066		
Number of menopausal symptoms	-0.533 (0.613)	-0.035	0.385		
Number of pelvic floor dysfunctions	-1.742 (0.905)	-0.077	**0.055**		

**Table 4:** Multiple linear regression analysis of light intensity physical activity as dependent variable.

	Light physical activity			model	
	B (SE)	Beta	p-value	R^2^	p-value
**Model 1**				0.019	**0.007**
Cumulative reproductive history index	3.845 (4.203)	0.037	0.361		
Number of menopausal symptoms	6.391 (2.747)	0.094	**0.020**		
Number of pelvic floor dysfunctions	7.789 (3.996)	0.077	**0.052**		
**Model 2: adjusted with BMI**				0.030	**0.001**
Cumulative reproductive history index	4.364 (4.189)	0.042	0.298		
Number of menopausal symptoms	6.335 (2.735)	0.093	**0.021**		
Number of pelvic floor dysfunctions	8.788 (3.996)	0.087	**0.028**		
**Model 3: as 2 and education**				0.073	**<0.001**
Cumulative reproductive history index	2.683 (4.109)	0.026	0.514		
Number of menopausal symptoms	6.459 (2.675)	0.095	**0.016**		
Number of pelvic floor dysfunctions	7.173 (3.920)	0.071	0.068		
**Model 4: as 3 and chronic diseases**				0.075	**<0.001**
Cumulative reproductive history index	2.624 (4.111)	0.025	0.524		
Number of menopausal symptoms	6.635 (2.686)	0.098	**0.014**		
Number of pelvic floor dysfunctions	7.635 (3.968)	0.076	**0.055**		
**Model 5: as 4 and smoking**				0.075	**<0.001**
Cumulative reproductive history index	2.861 (4.119)	0.027	0.488		
Number of menopausal symptoms	6.532 (2.689)	0.096	**0.015**		
Number of pelvic floor dysfunctions	7.650 (3.969)	0.076	**0.054**		

The number of menopausal symptoms was found to be significantly associated with light physical activity (**Table 4**). The association between pelvic floor dysfunction and light physical activity did not reach statistical significance in the fully adjusted model. Cumulative reproductive history index was also not significantly associated with light physical activity. The fully adjusted model explained 7.5% of the variation of time spent on light intensity physical activities.

## Discussion

Given the known health benefits of physical activity, effort must to be taken to promote physically active lifestyles. Therefore, it is important to identify the reasons for low physical activity. In this study, we focus on factors that are unique to middle-aged women. We found that 61% of Finnish women between the ages of 48 to 55 were inactive, defined as performing less than 150 min/week of moderate to vigorous physical activity. Among the lifestyle and biological factors we investigated in this study, BMI, smoking habits, reproductive history (namely gestations and parity), and pelvic floor dysfunctions differed between active and inactive women. Both menopausal symptoms and pelvic floor dysfunctions were commonly experienced by women in both groups, although only pelvic floor dysfunction differed among groups. We show that pelvic floor dysfunction was negatively associated with MVPA_10_ (p = 0.037), until controlling the model with chronic disease and smoking (p = 0.055). Perceived menopausal symptoms were positively associated with light physical activity (p = 0.015) while pelvic floor dysfunction (p = 0.054) and cumulative reproductive history index (p = 0.488) were not. To our knowledge, this is the first study to show a significant association between objectively measured intensities of physical activity and female reproductive factors in middle-aged women.

Current physical activity recommendations by the WHO are based on MVPA_10_, because presently there is the most evidence on health benefits of this type of physical activity [2]. Although light physical activity can also produce health benefits, it is less studied than MVPA_10_, possibly because light physical activity it is more difficult to measure. Subjective reports on physical activity mostly consider leisure-time activities, although occupational physical activity may contribute considerably to the total amount of physical activity. In the current study, we measured and described total daily amount of physical activity done at both light and moderate-to-vigorous intensities and included both leisure-time and occupational activities. Among our middle-aged women, working time comprised 25% of the total measured time on average. The use of objective physical activity measurement in this study allowed us to further investigate the time spent on MVPA_10_ and on light physical activity. Light physical activity ranged from 450 to 2690 cpm of tri-axial vector magnitude measures, corresponding to the commonly used 100 and 2020 cpm of vertical axis data [26]. To put these cpm ranges in further perspective, light physical activity intensity is considered to be 1.5–3 metabolic equivalent time and includes low intensity activities such as slow speed walking or bicycling.

In total, 39% of the middle-aged Finnish women participating in this study were categorized as physically active based on the WHO physical activity recommendation, theoretically indicating that their physical activity levels garner health benefits. In our data, there were no significant differences in education, marital status, or employment status between physically active and inactive women. This is not consistent with previous studies that, reported low education and low income to be associated with reduced participation in physical activities [27–29]. One reason for this discrepancy may be that our measure of socioeconomic status was simplistic, as all socioeconomic factors were conceptualized as dichotomous variables. However, using education, for example, as a trichotomized variable did not change the result (the distributions in active vs. inactive groups were 2.0% vs. 2.5% for primary, 57.0% vs. 59.1% for secondary, and 41.0% vs. 38.4% for tertiary level education). Not finding significant associations may be related to the fact that, the level of education in women is high in Finland [28], and having only little variation among education levels lacks the statistical power to differentiate any association between active and inactive women. A recent meta-analysis concluded that there are too few studies to determine if marital status is a determinant of physical activity [30]. Our findings regarding lifestyle factors are consistent with results from previous studies [27,29], indicating that physically active women have healthier body composition based on BMI and that they are less often smokers compared with inactive women.

Few studies have investigated associations between physical activity and parity or gestations especially among middle-aged women. Steindorf et al. found that nulliparous women who were over 50 years old were less physically active than age-matched parous women [31]. We also found significant differences in the number of gestations and parity between active and inactive women. However, in our study, physically active women were more often nulliparous and without pregnancies, less often had one to two pregnancies or birth, and more often had three or more gestations and high parity compared with inactive women. It is possible that the differences in physical activity measurement (subjective versus objective) or population health may underlie the discrepancy between previous reports and our results. Our study population was relatively healthy; therefore, health issues should not prevent engagement in physical activity. However, we do not know if our study participants were nulliparous or multiparous either by choice or due to differences in fecundity. The higher engagement in physical activity for women with several children may be a reflection of becoming accustomed to a more active lifestyle at younger ages when being responsible for children and a larger household, as was suggested previously by Steindorf et al. [31].

Although the difference between active and inactive groups was not statistically significant (p = 0.063), almost twice as many women in the physically active group had a cumulative reproductive history index value of 3–4 compared to the physically inactive group (10.4% vs. 5.7%) indicating that higher index values may associate with higher tendency to be active. The components of the cumulative reproductive history index are linked. Thus, it can be speculated that the components together indicate a greater tendency for parenthood, which requires an active lifestyle at least when the children are young. These early experiences could promote the maintenance of physical activities later in life, and especially when children are older and the mothers have more time and freedom to choose their own activities. Though miscarriages can be interpreted as a negative health factor, one to two miscarriages are not abnormal, and nearly 20% of all pregnancies end at early onset [32]. Thus, women who have more pregnancies are also more likely to experience a miscarriage. Therefore, the cumulative reproductive history index may in general be an indicator of reproduction, which may have a positive association with an active lifestyle. However, this is highly speculative since we have not investigated associations among the different components of the cumulative reproductive history index and physical activity.

Menopausal symptoms were commonly experienced in our middle-aged population. Nearly 80% of the women reported to have some symptom, with the most common being vasomotor (~60%) and psychological (~50%) symptoms. The rates are higher than reported in previous studies [16,33,34], potentially because those studies focused on certain menopausal stages [33], bothersome symptoms [16], or hormone therapy usage [34], while our study included all 48 to 50-year-old women regardless of their menopausal status and excluded women undergoing hormone therapy. Furthermore, we did not consider the perceived annoyance of menopausal symptoms. Despite the high number of women reporting menopausal symptoms, the prevalence of these symptoms did not differ between physically active and inactive groups, but was instead found to be significantly associated with greater light physical activity. Moilanen et al. reported physically active women as having less somatic menopausal symptoms than inactive women [16]. To date, several exercise trials targeted to treat vasomotor menopausal symptoms have been performed, but the results are inconclusive (summarized in a recent Cochrane review: [35]). We attempted to investigate if experiencing menopausal symptoms is associated with low physical activity rather than if physical activity could treat menopausal symptoms. We found that women with a higher cumulative number of self-reported menopausal symptoms spent more time engaging in light physical activity, while there was no association with MVPA_10_. Additional longitudinal studies are needed to determine if middle-aged women experiencing menopausal symptoms are restricted or unwilling to perform higher intensity physical activities and therefore spend more time on light intensity physical activity.

An important finding of the current study was that pelvic floor dysfunction was less prevalent among physically active than inactive women. The most common type of pelvic floor dysfunction was stress urinary incontinence, which occurred in 32% of physically active women and 43% of inactive women. Considering all types of pelvic floor dysfunctions, 44% of active and 60% inactive women reported a dysfunction while ~20% of all women had two or more types of pelvic floor dysfunction. Previous research has shown that age, white race, greater parity, higher BMI, and lower levels of physical activity are associated with greater odds of urinary incontinence [36,37]. Another study did not find any association between pelvic organ prolapse and lifetime physical activity, but showed that middle-aged women with pelvic organ prolapse had greater BMI and parity [38]. We found a negative association between MVPA_10_ and pelvic floor dysfunction (Table 3, models 1–3), which diminished after adjustment for chronic diseases (p = 0.055). This may indicate that chronic diseases are associated with pelvic floor dysfunction in addition to MVPA_10_. Our study did not measure how harmless or harmful the reported pelvic floor symptoms were considered by the women, which may also have influenced the magnitude and significance of the associations that were measured. Furthermore, we cannot know without further studies if women experiencing more bothersome symptoms of pelvic floor dysfunction would be more restricted from performing high intensity activities than women with less bothersome symptoms.

A major strength of our study is the objective measurement of daily physical activity in 647 compliant middle-aged women, allowing a thorough evaluation of the time they spent doing physical activities at low to vigorous intensities. Acceptable accelerometer data was obtained from 92% of study participants with the mean number of accelerometer-wearing days of 6.8 out of 7 days and mean wearing time of over 15 hours per day with no differences in compliance between physically active and inactive women. Previous studies have reported lower achievable and acceptable compliance (e.g., 60% of participants providing valid data over 10 hours per day for 4 days [39]). The primary limitation of our study is its cross-sectional nature, which presents us from providing estimates of causality. There are other limitations to this study as well. Menopausal symptoms and pelvic floor dysfunction information were self-reported, which may have resulted in some misclassifications. Furthermore, severity of the symptoms was not investigated. However, the determination of menopausal status with menstrual diary and hormonal assessments was accurate, and we considered simultaneously different types of pelvic floor dysfunction and menopausal symptoms. Our study population comprised of a narrow age range of relatively typical and healthy Finnish women, thereby providing a quite homogenous study group. This can be considered both a strength and a limitation. This type of population sample permits precise measurements without a need to control heterogeneity of differences in age, race, or health and provides good estimates for Nordic and other mainly Caucasian middle-aged female populations. On the other hand, our results may not be generalizable to more heterogenic western populations. Finally, we did not control for environmental factors, which may also contribute to physical activity behavior, but measurement times were evenly distributed around the four seasons, thus controlling seasonal variation in physical activities.

With these limitations in mind, we conclude that both menopausal symptoms and pelvic floor dysfunction are commonly experienced among middle-aged women and are associated with different physical activity levels. In line with earlier studies [29,40], an alarmingly high percentage (61%) of middle-aged women did not meet the physical activity recommendation by the WHO. Inactive women reported more pelvic floor dysfunctions than active women, and the perceived number of pelvic floor dysfunction was associated with lower MVPA_10_. In addition, the perceived number of menopausal symptoms was associated with greater light physical activity. Although our models were able to explain only a small portion of total physical activity (6–7.5%), it is not meaningless in a larger context of promoting physical activity. For instance, keeping other mediating factors constant, the model predicts that a one unit increment in the perceived pelvic floor dysfunction leads to a 1.7 min decrement in MVPA_10_. If a person has a low level MVPA_10_ to start with, a ~2-minute reduction in daily activity may represent a substantial reduction when occurring over months and years. Specifically, in our data, 1.7 minutes was almost 19% of the mean MVPA_10_ performed by the women belonging to the inactive group. This study, therefore, indicates that sex-specific factors such as having menopausal symptoms and pelvic floor dysfunction should be identified and considered in physical activity promotion. For instance, being aware of the condition of the pelvic floor when promoting physical activity for a middle-aged woman may enable the health care provider to aid women in achieving health-enhancing amounts of physical activity.

## Supporting information

S1 Table Comparison between the vertical axis and the tri-axial vector data. (DOCX)

S2 Table Comparisons of tri-axial and vertical axis analysis for different physical activity intensities. (DOCX)

S3 Table Effect of potential confounding factors to the regression coefficient. (DOCX)
