# Supplementary material for: Female reproductive factors are associated with objectively measured physical activity in middle-aged women
Source: PLoS One. 2017 Feb 22;12(2):e0172054. doi: 10.1371/journal.pone.0172054 (PMC5321412; doi:10.1371/journal.pone.0172054)
Supplement: S1 Table — (DOCX) [file pone.0172054.s001.docx]

**S1 Table.** Comparison between the vertical axis and the tri-axial vector data.

|  | Mean difference from sedentary time with vertical axis cut-point 100 cpm | Pearson correlation coefficient (r) | p-value |
| --- | --- | --- | --- |
| VM3 cut-point 200 cpm | -76.63 | 0.93 | <0.001 |
| VM3 cut-point 300 cpm | -43.14 | 0.95 | <0.001 |
| VM3 cut-point 400 cpm | -15.21 | 0.96 | <0.001 |
| VM3 cut-point 450 cpm | -2.22 | 0.96 | <0.001 |
| VM3 cut-point 500 cpm | 10.38 | 0.96 | <0.001 |
| VM3 cut-point 600 cpm | 34.83 | 0.96 | <0.001 |

Tri-axial vector magnitude (VM3) was computed as composite vector magnitude of three individual orthogonal planes to accommodate VM3 cut-points to be able to distinguish time spent on different activity intensities [1]. Since to our knowledge sedentary cut-point for adults’ VM3 have not been published, we computed sedentary time from the vertical axis with the widely used 100 cpm as cut-point and then compared to sedentary time analyzed from the VM3 data with several cut-points by using all valid daily accelerometer data obtained with both VM3 and vertical axis analysis including 6284 measurement days. On the other hand, 100 counts per minute (cpm) is widely used cut-point in vertical axis only analyses. Therefore, sedentary time was computed from the vertical axis with the widely used 100 cpm and then compared to sedentary time analyzed from the VM3 data with several cut-points (Table S1). Sedentary time calculated with cut-point 450 cpm correlated highly to 100 cpm in vertical axis analyses (S1 Table) and was chosen to supplement Sasaki et al. (2011) VM3 cut-points (i.e. light >450 to ≤2690 cpm; moderate >2690 to ≤6166 cpm; vigorous >6166 cpm). Supporting information Table S2 shows mean difference and Pearson correlation coefficient between VM3 and vertical axis data for all different physical activity intensity modalities from sedentary to vigorous intensities. Mean time spent on each intensity level during measurement days is calculated using our final data with 647 middle-aged women.

References

1. Sasaki JE, John D, Freedson PS. Validation and comparison of ActiGraph activity monitors. J Sci Med Sport. 2011;14: 411-416.
