# Supplementary material for: Female reproductive factors are associated with objectively measured physical activity in middle-aged women
Source: PLoS One. 2017 Feb 22;12(2):e0172054. doi: 10.1371/journal.pone.0172054 (PMC5321412; doi:10.1371/journal.pone.0172054)
Supplement: S2 Table — (DOCX) [file pone.0172054.s002.docx]

**S2 Table.** Comparisons of tri-axial and vertical axis analysis for different physical activity intensities.

|  | Mean time (min) | Mean difference | Pearson correlation coefficient (r) | p-value |
| --- | --- | --- | --- | --- |
| VM3 sedentary time  (≤450 cpm) | 570.65 |  |  |  |
| Vertical axis sedentary time  (≤100 cpm) | 573.89 | -3.24 | 0.967 | <0.001 |
| VM3 light PA  (>450 to ≤2690 cpm) | 294.75 |  |  |  |
| Vertical axis light PA  (>100 to ≤2020 cpm) | 301.85 | -7.10 | 0.946 | <0.001 |
| VM3 moderate PA  (>2690 to ≤6166 cpm) | 44.50 |  |  |  |
| Vertical axis moderate PA  (>2020 to ≤5999 cpm) | 32.78 | 11.73 | 0.834 | <0.001 |
| VM3 vigorous PA  (>6166 to <25000 cpm) | 5.27 |  |  |  |
| Vertical axis vigorous PA  (>5999 to <20000 cpm) | 1.99 | 3.27 | 0.802 | <0.001 |
| VM3 too high, spurious PA  (>25000 cpm) | 0.0013 |  |  |  |
| Vertical axis too high, spurious PA  (>20000 cpm) | 0.0022 | -0.0009 | 0.954 | <0.001 |

In the multiple linear regression equations (Y= B_0_ + B_1a_X_a_ + B_1b_X_b_ + B_1c_X_c_ + B_2_X_2_) for light intensity physical activity (light PA) and moderate to vigorous intensity physical activity accumulated in the bouts of at least 10 minutes (MVPA_10_), B_1a,_ B_1b_ and B_1c_ are the estimated regression coefficients that quantifies the association between the independent risk factors; cumulative reproductive history index (X_a_), menopausal symptoms (X_b_) and pelvic floor dysfunction (X_c_) and the outcome (light PA or MVPA_10_) adjusted for potential confounder X_2_ and B_2_ is the estimated regression coefficient that quantifies the association between the potential confounder and the outcome. We tested each potential confounder (education, marital status, employment status, BMI, chronic diseases, smoking, alcohol consumption) one-by-one (Table S3) in the light PA and MVPA_10_ models. According to [1] the ones causing at least 9% change in at least one of the regression coefficient (B_1a,_ B_1b_ or B_1c_) of the independent risk factors were considered to be confounders and were thus included into the final models presented in the article Tables 3 and 4. Variable was added into the both models if it was found to be a confounder in either one of the models to ease the comparison of the results for in either light PA or MVPA_10_.

References

1. Kleinbaum D, Kupper LL, Muller KE. *Applied Regression Analysis and Other Multivariable Methods*. 2nd edition ed. Boston: PWS-Kent; 1988.
