# Supplementary material for: Female reproductive factors are associated with objectively measured physical activity in middle-aged women
Source: PLoS One. 2017 Feb 22;12(2):e0172054. doi: 10.1371/journal.pone.0172054 (PMC5321412; doi:10.1371/journal.pone.0172054)
Supplement: S3 Table — (DOCX) [file pone.0172054.s003.docx]

**S3 Table.** Effect of potential confounding factors to the regression coefficient.

|  |  | | **Education** | |  | **Marital status** | |  | **Employment status** | |  | **BMI** | |  | **Chronic diseases** | |  | **Smoking** | |  | **Alcohol consumption** | |
| --- | --- | --- | --- | --- | --- | --- | --- | --- | --- | --- | --- | --- | --- | --- | --- | --- | --- | --- | --- | --- | --- | --- |
|  | B_without conf._ | | B_with conf._ | **ΔB-%** |  | B_with conf._ | **ΔB-%** |  | B_with conf._ | **ΔB-%** |  | B_with conf._ | **ΔB-%** |  | B_with conf._ | **ΔB-%** |  | B_with conf._ | **ΔB-%** |  | B_with conf._ | **ΔB-%** |
| **Light PA** |  |  | |  |  |  |  |  |  |  |  |  |  |  |  |  |  |  |  |  |  |  |
| cumulative reproductive history index | 3.84 | 2.16 | | **-78.2** |  | 3.98 | 3.4 |  | 3.85 | 0.13 |  | 4.36 | **11.9** |  | 3.78 | -1.7 |  | 3.91 | 1.6 |  | 3.81 | -1.1 |
| menopausal symptoms | 6.39 | 6.52 | | 1.9 |  | 5.89 | -8.5 |  | 6.41 | 0.27 |  | 6.34 | -0.9 |  | 6.61 | 3.3 |  | 6.37 | -0.4 |  | 6.81 | 6.1 |
| pelvic floor dysfunction | 7.79 | 6.11 | | **-27.4** |  | 7.38 | -5.5 |  | 7.80 | 0.10 |  | 8.79 | **11.4** |  | 8.37 | 6.9 |  | 7.81 | 0.2 |  | 7.59 | -2.6 |
| **MVPA_10_** |  |  | |  |  |  |  |  |  |  |  |  |  |  |  |  |  |  |  |  |  |  |
| cumulative reproductive history index | 1.37 | 1.45 | | 6.1 |  | 1.36 | -0.7 |  | 1.38 | 0.73 |  | 1.59 | **14.1** |  | 1.35 | -1.3 |  | 1.51 | **9.2** |  | 1.36 | -0.3 |
| menopausal symptoms | -0.51 | -0.52 | | 1.2 |  | -0.48 | -6.2 |  | -0.48 | -6.65 |  | -0.54 | 4.5 |  | -0.45 | **-13.1** |  | -0.56 | **9.4** |  | -0.49 | -3.2 |
| pelvic floor dysfunction | -2.36 | -2.26 | | -3.8 |  | -2.28 | -3.8 |  | -2.35 | -0.64 |  | -1.93 | **-22.4** |  | -2.21 | -7.2 |  | -2.33 | -1.3 |  | -2.34 | -0.9 |

BMI= body mass index, B= regression coefficient, ΔB-% = change percentage of the regression coefficient calculated as

(B_with confounder_-B_without confounder_)/B_with confounder_ x 100
